# The Interplay of COVID 19, Remote Contact, and Race as Risk Factors for Depression

**DOI:** 10.1093/geroni/igab046.2677

**Published:** 2021-12-17

**Authors:** Youngjoon Bae, Katherine Abbott, Mark Pachucki

**Affiliations:** 1 University of Massachusetts, Amherst, Amherst, Massachusetts, United States; 2 Miami University, Oxford, Ohio, United States

## Abstract

There have been concerns about how social distance policies and lockdowns due to COVID-19 have affected loneliness and depression among older adults in ways that may magnify racial disparities in health. We conducted panel logistic regression analyses with random effects using national data spanning 2004 to 2016 and the COVID-19 module (Wave 2020, administered in June and September) from the Health & Retirement Study (n=15,504). Individuals living in a nursing home, diagnosed with dementia or Alzheimer’s disease, and 64 years of age or younger were excluded from analyses. Age, gender, Hispanic status, education, marital status, working status, wealth, BMI, physical activity, smoking, drinking, and difficulty in meal preparation, eating without help, and grocery shopping were included as control variables. Findings suggest that older adults did not appear to experience increased loneliness during the pandemic relative to prior waves. However, Wave 2020 was an independent risk factor for depression. Greater in-person contact (OR: 0.97, CI: 0.95-0.99, p-val: 0.001) and remote contact (OR: 0.99, CI: 0.97-0.996, p-val: 0.008) were each independently associated with slightly decreased depression. Older Black Americans tended to be more depressed than their White counterparts (OR: 1.50, CI: 1.20-1.86, p-val: <0.001). However, a null interaction between race and wave suggested that Black Americans did not experience more increased depression in 2020 relative to prior waves. Analyses suggest that the COVID-19 pandemic might change at-risk groups for depression and communication by remote technology – often considered an inferior but necessary stopgap measure. Implications for practice and policy will be discussed.

